# A Description of COVID-19 Lifestyle Restrictions Among a Sample of Rural Appalachian Women

**DOI:** 10.13023/jah.0301.02

**Published:** 2021-01-24

**Authors:** Michele Staton, Martha Tillson, J. Matthew Webster

**Affiliations:** University of Kentucky

**Keywords:** Appalachia, COVID-19, rural women, social isolation

## Abstract

**Background:**

COVID-19 has led to swift federal and state response to control virus transmission, which has resulted in unprecedented lifestyle changes for U.S. citizens including social distancing and isolation. Understanding the impact of COVID-19 lifestyle restrictions and related behavioral risks is important, particularly among individuals who may be more vulnerable (such as rural women with a history of substance use living in Appalachia).

**Purpose:**

The overall purpose of this study was to better understand the perceptions of lifestyle changes due to COVID-19 restrictions among this vulnerable group.

**Methods:**

The study included a mixed methods survey with a convenience sample of rural women (n=33) recruited through a closed, private Facebook group.

**Results:**

Study findings indicated that COVID-19 restrictions related to limited social activities and interactions with family and friends had a significant impact on women.

**Implications:**

Findings suggest that social isolation may have a number of unintended consequences for rural women, and implications for rural health practitioners are discussed.

## INTRODUCTION

COVID-19, the disease caused by the severe acute respiratory syndrome (SARS) coronavirus, has been declared a global pandemic by the World Health Organization.^1^ The infection rate grew exponentially with more than 20 million cases worldwide and more than 736,000 deaths across more than 200 countries as of August 2020.^2^ The infection rate is high because the virus spreads quickly through person-to-person contact through contaminated droplets, hands, or surfaces.^1^ COVID-19 is characterized by symptoms that mimic the flu including fever, cough, and fatigue; however, the death risk considerably increases with pre-existing health issues.^3^

Because there is no vaccine or effective treatment as of late 2020, public health efforts have primarily focused on reducing virus transmission. Widespread U.S. restrictions have included social distancing, self-isolation, travel restrictions, and closing of schools, restaurants, and businesses.^4^ While limited research has shown effectiveness in reducing transmission,^5^ lifestyle changes and social restrictions also increase behavioral health risks including mental health concerns^6^ and illicit drug use.^7^ Research has highlighted the prevalence of mental health issues (depression, anxiety, and psychological distress) among individuals in quarantine or isolation related to the COVID-19 pandemic.^8^ Research is limited for changes in illicit drug use as a result of COVID-19 restrictions, but one study recently reported signals in the state’s emergency medical services database for a significant increase in opioid overdoses.^9^

Most of the recent COVID-19 research focuses on secondary data analysis, often conducted in urban areas of the U.S. and internationally, neglecting populations of individuals who may be adversely impacted by COVID-19 restrictions. In general, the rate of COVID-19 infection has been less in rural Appalachian communities.^10^ However, because of higher rates of underlying chronic diseases, limited specialty healthcare, increased rates of poverty, and limited transportation, individuals in rural Appalachian communities may be more vulnerable.^11^ In addition, social restrictions and isolation may have more of a profound impact on rural Appalachians who rely on close family and friend networks for social support. Rural Appalachian women, in particular, may be more vulnerable due to increased family and childcare responsibilities and stress related to family health.^12^ In addition, women with a history of substance use may rely more heavily on family and friend networks to sustain recovery efforts.

Although the effects of COVID-19 have yet to be fully realized, it is critical to begin to understand the pandemic’s impact on different groups, particularly those who have long-standing health disparities and are at increased vulnerability due to other behavioral health needs (such as substance use).

Because rural Appalachian women experience numerous health and behavioral health vulnerabilities, a better understanding of the impact of COVID-19 restrictions among this population is critical for the field. This study provides an exploratory look at the perceptions of the impact of COVID-19 restrictions among rural Appalachian women using mixed methods and describes the perceptions of substance use and mental health changes in the community during the pandemic.

## METHODS

### PARTICIPANTS

This convenience sample (N=33) included rural Appalachian women who previously participated in a larger federally-funded trial (2012–2018) focused on substance use and associated behavioral risks. By nature of involvement with that study,^13^ women in the current analysis had a history of justice-involvement and substance use. However, they were all living in the community at the time of the current study data collection.

### MEASURES

#### Demographics

Demographics included age (age at the time of the interview), race (white vs. non-white), health (description of health, doctor’s visits, medications), housing (living situation in the past month), employment (current employment status), and children (currently have children, living arrangement).

#### COVID-19 restrictions

Quantitative measures were developed to assess the impact of state and federal guidelines for reducing COVID-19 transmission. Measures were grounded in the time frame of “the past month”, which coincided with implementation of state restrictions initiated in March 2020. Participants were also asked how frequently they had engaged in activities in the past month such as visiting local businesses, family, and friends (categorical responses of “never,” “rarely,” “sometimes,” or “often”). Qualitative measures focused on participants’ responses to a single open-ended question, “How has the coronavirus impacted your life the most?” Answers were transcribed verbatim by interviewers.

##### Substance use

Participants were asked about noticeable changes in drug and alcohol use in the community and among family members in the past month. They were also asked about past month use of alcohol (yes/no) and illicit drug use (yes/no), as well as their perception of whether or not their use was a change in their use pattern. Participants who reported past-month use were also asked questions from the Alcohol Use Disorders Identification Test-Concise (AUDIT-C)^14^ to assess hazardous drinking (score of 0 – 12, higher scores indicative of more risky drinking) and the CAGE-AID, a brief 4-item screen to assess risky drug use.^15^

#### Mental health

Mental health was assessed using the Patient Health Questionnaire (PHQ-9)^16^ which provides a score indicative of depression symptoms.

### PROCEDURE

Study recruitment was conducted using a closed, private Facebook group, established to maintain contact with women who participated in a larger trial focused on behavioral health.^13^,^17^ All women in the Facebook group were considered eligible to participate in this COVID-19 survey focused on rural women. At the time of study recruitment, 198 women were “friends” on the closed site, but those profiles were not verified for recent activity. A flyer was posted on the study Facebook site for 2 months, and interested participants were provided study contact information to schedule an appointment. Interested participants were invited to call in to the designated study phone line. The survey was preceded by an informed consent script that included an overview of the purpose of the study, confidentiality, and use of the data. Consenting participants provided verbal consent due to the phone-based survey, a process approved by the university IRB. Interview questions were read to the participant over the phone and responses were recorded in a computer-based Research Electronic Data Capture (REDCap) survey format. The survey took approximately 20 minutes to complete, and participants were paid $15 for their time. All data were collected between April 22, 2020 and June 22, 2020.

### ANALYTIC PLAN

Univariate descriptive statistics were calculated for all quantitative measures. For qualitative data, two of the study authors identified and discussed key themes across responses to the open-ended question. These themes were compiled into a codebook of five main codes, which were then applied to the data. Results were interpreted using an explanatory mixed methods design,^18^ in which qualitative analysis was used to expand and elaborate upon quantitative findings. Summaries of key themes and selective illustrated quotes are presented.

## RESULTS

### SAMPLE DESCRIPTION

Participants were about 35 years old (SD=6.03) and 100% white (see Table 1). About half (51.5%) had visited a doctor in the past month, with more participants utilizing in-person (n=10) versus telehealth (n=7) services. Approximately three-fourths (72.7%) of women reported taking some form of prescription medication, including medications for opioid use disorder (MOUD). Most participants were housed either in their own home (66.7%) or someone else’s (27.3%), with more than half reporting living with a partner (54.5%) and/or in a household with children (57.6%). Less than half of participants (39.4%) reported being currently employed. Almost all participants (93.9%) had children of their own, and more than half (54.5%) had their children living with them.

### PERCEIVED CHANGES IN SUBSTANCE USE AND MENTAL HEALTH

About a quarter of participants said they had noticed a change in friends’ or family’s use of alcohol in the past month (24.2%) or had noticed an increase in drug use locally since COVID-19 began (27.3%). One participant (3.0%) reported past-month alcohol use and five (15.2%) reported past-month use of any illicit drug. Participants’ scores on the PHQ-9 ranged from 0 to 20, with an average of 6.79 (*SD*=5.88), translating to “mild” depression symptomology. [Table t2-jah-3-1-4]

### QUANTITATIVE ANALYSIS OF COVID-19 IMPACT

Almost all participants reported that COVID-19 had impacted their lifestyle, with responses ranging from a little bit (54.5%) to a major way (42.4%), though only 12.1% knew someone who had tested positive for the virus (no participants had tested positive at the time of their interview). Participants’ thoughts on social distancing varied, with 48.5% reporting it was “easy” or “extremely easy,” and 39.4% saying it was “difficult” or “extremely difficult.”

Of participants who reported being employed (n=13), almost half (46.2%, or n=6) said they had not been affected by changes due to COVID-19. The most common work-related impact cited was scheduling, such as working more hours or switching shifts. More than three-fourths of participants who reported living with their children (77.8% of n=18) indicated that COVID-19 had not affected their childcare situation. [Table t3-jah-3-1-4]

Participants also reported daily activity restrictions. Describing the past month, most indicated that they had not attended social events such as a party (97.0%), church (90.9%), or visited with friends in the friends’ home (78.8%). In addition, none of the study participants reported attending in-person AA/NA meetings. However, modal responses for other activities suggested that participants most frequently reported going to a store like Walmart “rarely” (36.4%), going to the grocery store “sometimes” (42.4%), and going outside for a walk or exercise “often” (57.6%).

### QUALITATIVE ANALYSIS OF COVID-19 IMPACT

Qualitative analysis focused on the narrative of individual experiences related to the impact of COVID-19 restrictions, which generally referenced five primary themes. The most common theme, mentioned by 48.5% of participants, was *restricted mobility*, including going out for shopping, recovery support meetings, or fun. Participants expressed frustration at “not being able to leave home or go anywhere,” or not being able to bring children or partners into stores with them. However, these restrictions did not seem grievously disruptive – participants used phrases such as “everything is a hassle,” or “it’s just aggravating”. Secondly, about a third of participants (30.3%) discussed *restricted social interaction*, such as not being able to see family or friends face-to-face. While mentioned by fewer participants, this seemed to have a greater impact, as they had seen family “all the time” or “almost daily before all of this started happening.” Restricted visits in hospitals were especially difficult for two participants who had just given birth and one participant whose fiancé was “on life support and in the hospital,” who found themselves separated from their loved ones under particularly stressful circumstances. The third most prevalent theme was *finances or work situation* (28.1% of participants), such as working different shifts, working fewer hours or more hours (due to other employees being laid off), not working at all (particularly for self-employed participants), and general financial struggles. The last two themes were each mentioned by 15.6% of participants. These included *concern for health* – whether health of family members, friends, or the participant themselves – and *mental health*, including increased stress or worry. A few participants mentioned experiencing “a lot of stress,” one said that the situation had “increased her anxiety,” and another stated that she was “afraid to go anywhere or do anything.”

## IMPLICATIONS

The emergence of COVID-19 necessitated swift federal and state response to control virus transmission, which has resulted in an unprecedented impact on the lifestyles of U.S. citizens including social distancing, self-isolation, and travel restrictions^4^. Understanding the impact of COVID-19 restrictions and related behavioral risks is critically important, particularly among individuals who may be more vulnerable – such as rural Appalachian women. The overall purpose of this study was to better understand the perceptions of the impact of COVID-19 restrictions among rural Appalachian women with a history of substance use.

The challenges associated with social isolation are likely even more pronounced in rural areas where there is increased reliance on family and friends for support.^19^ Findings indicated that the majority of women in the sample reported not attending social activities (including self-help groups like AA or NA), and rarely going out for activities like grocery shopping. This sense of restricted mobility seemed to be conceptualized as frustration rather than distress in the qualitative analysis. However, the bigger impact seemed to be related to restricted social interaction and not being able to visit with family and friends, which were viewed as more stressful. Women in the study mentioned seeing family “all the time” prior to implementation of COVID-19 restrictions. Being separated from family was further challenging if there were health concerns among either the participant or the family member. While COVID-19 restrictions related to social interaction are difficult in general, they appear to be particularly stressful for rural women who rely heavily on social networks for support.^19^,^20^

While rural Appalachian women may be at increased risk due to social isolation, those who have a history of substance use may be even more vulnerable. The convenience sample in this study included women with a substance use history. The research literature on women and substance use is replete with examples of how drug use patterns are closely tied to women’s relationships,^21^ and times of isolation and loneliness may be high-risk for relapse.^22^ However, findings from this study indicated that only a small percentage of women reported relapse to drug use during the study time frame, and most reported positive indicators of sustained recovery including stable housing and living with children. In addition, only about a quarter reported perceptions of increased drug use locally among community members. Findings from this study may suggest that even during the challenging and stressful times of a pandemic, future research should examine resilience among rural women in avoiding relapse.

One of the biggest concerns around the unintended consequences of COVID-19 restrictions is mental health.^8^ Women in this study reported PHQ-9 symptom profiles consistent with “mild” depression, and mental health symptoms were articulated as “increased anxiety” and “a lot of stress”. This is consistent with other studies of rural women experiencing depression during periods of social isolation and loneliness.^23^ This is an important area for behavioral health providers in developing and targeting interventions for unique and vulnerable women during times of increased crisis.

This study has limitations. Recruitment involved a convenience sample of rural Appalachian women who joined a private, closed Facebook page following participation in a larger parent study. A recruitment notification was added to the study “newsfeed”, but the number of possible participants who remained active on the site who might have saw the post was not determined. While studies have shown that Facebook is a viable platform to remain in contact with study participants,^17^ women also change their profiles often and delete accounts. Therefore, generalizability of findings is limited due to the small number of rural women who participated. The small sample size also limited more complex analysis. An additional limitation is that data was self-reported, and the extent to which state COVID-19 guidelines were followed (both at the individual level and the community level) could not be verified. The data collection window was also limited to 2 months, which may not have fully captured the full range of COVID-19 lifestyle restrictions nor the potential for increasing numbers of positive cases over time. This is particularly relevant since COVID-19 cases were fairly limited in this rural region of Appalachia at the time of data collection, but at the time of this manuscript preparation, were rising in some targeted areas. We also recognize that the measure did not assess traditional activities prior to the implementation of COVID-19 restrictions, so measuring changes in behavior was not possible.

Despite these limitations, this study makes an important contribution to the empirical literature by increasing our understanding of the impact of COVID-19 lifestyle restrictions on rural women in Appalachia. Most of the research to date is based on secondary data analysis and systematic reviews, many of which focus on larger urban samples. Social connections to family and friends are salient in rural Appalachia and critical for support, particularly for individuals in recovery.

When interaction with family and friends are restricted, even if the restrictions are warranted to reduce virus transmission, there may be other health or mental health consequences. Appalachian women with a history of health or behavioral health issues may be even more vulnerable. Study findings highlight the importance for health professionals to be attentive to the unique needs of rural women and for the development of health and behavioral health interventions to deal with the unintended ramifications of COVID-19. Specifically, rural Appalachian health care providers should screen for mental health and substance use issues for all patients and help them connect with appropriate resources as needed. Furthermore, providers might consider contacting existing patients to provide COVID-19 prevention education, to remind them of available health care and community resources, and to stress the importance of continuing to safely engage with others to avoid feelings of isolation. Such efforts could help reduce COVID-19 transmission in rural Appalachia and also mitigate pandemic-related problems being experienced by those who have not contracted the virus.

**SUMMARY BOX**

**What is already known about this topic?** Precautions to reduce the spread of COVID-19 have led to unprecedented lifestyle changes related to social distancing and social isolation. While early signals indicate that these lifestyle changes have been effective in reducing virus transmission, research is needed on the unintended consequences of these restrictions in rural communities.

**What is added by this report?** This report is the first to look at the impact of COVID-19 lifestyle restrictions on the health and behavioral health of rural Appalachian women. Among rural women, who depend heavily on family and friend networks, COVID-19 precautions may have unintended consequences such as mental health issues.

**What are the implications for future research?** Findings highlight the importance for health professionals to be attentive to the unique needs of rural women and for the development of health and behavioral health interventions to address the unintended ramifications of COVID-19. Rural practitioners may also consider screening for mental health and substance use issues for all patients to help them connect with appropriate resources as needed. Providers may also consider contacting existing patients to provide COVID-19 prevention education, to remind them of available health care and community resources, and to stress the importance of continuing to safely engage with others to avoid feelings of isolation.

## Figures and Tables

**Table 1:** Descriptive Profile (N=33)

	%/Mean	n	SD	Range
**Demographics**				
Age in years	34.91		6.03	27–52
Race (% white)	100%	33		
**Health**				
How would you describe your health?	3.21 (Good)		1.22	1–5
Visited a doctor in the past month	51.5%	17		
In-person	30.3%	10		
Telehealth	21.2%	7		
Taking prescription medications (including MOUD)	72.7%	24		
Had to visit doctor to get prescriptions filled	39.4%	13		
**Housing**				
Where have you been staying in the past month?				
Your home	66.7%	22		
Someone else’s home	27.3%	9		
Hotel	3.0%	1		
Hospital	3.0%	1		
Who have you been staying with?				
Child(ren)	57.6%	19		
Partner	54.5%	18		
Other family members	18.2%	6		
Parents	12.1%	4		
Friends	3.0%	1		
**Employment**				
Currently employed	39.4%	13		
How many hours a week do you usually work? (*n*=13)	34.77		12.58	0–48
**Children**				
Has children	93.9%	31		
Has own children living with them	54.5%	18		

**Table 2 t2-jah-3-1-4:** Perceived and Self-reported Substance Use and Mental Health (N=33)

	%/Mean	N	SD	Range
**Perceptions of Use**				
Change in friends’/family’s past-month alcohol use	24.2%	8		
Noticed increase in local drug use since coronavirus	27.3%	9		
**Self-reported Use**				
*Used alcohol in the past month*	3.0%	1		
Change in past-month use (*n*=1)	Increased (100%)	1		
AUDIT-C Score (*n*=1; scale of 0–12)	7.00		-	-
*Used drugs in the past month*	15.2%	5		
Used most often:				
Suboxone	6.1%	2		
Marijuana	3.0%	1		
Methamphetamines	3.0%	1		
Methadone	3.0%	1		
Change in drug use in the past month (*n*=5)				
Increased	20.0%	1		
Stayed about the same	60.0%	3		
Decreased	20.0%	1		
CAGE-AID Score (*n*=5; scale of 0–4)	1.80		1.10	0–3
**Mental Health**				
PHQ-9 Score (scale of 0–27)	6.79		5.88	0–20

**Table 3 t3-jah-3-1-4:** COVID-19 Impact (N=33)

	%	n
**How much has COVID-19 impacted your lifestyle (past month)?**		
Not at all	3.0	1
A little bit	54.5	18
In a major way	42.4	14
**Self or anyone you know tested positive?**	12.1	4
Self	0.0	0
Family member	3.0	1
Friend	9.1	3
**How difficult or easy has social distancing been?**		
Extremely easy	12.1	4
Easy	36.4	12
Neither difficult nor easy	12.1	4
Difficult	30.3	10
Extremely difficult	9.1	3
**If employed (** ***n*** **=13), how has the coronavirus affected your work?**		
Not affected	46.2	6
Working more hours or switching shifts	23.1	3
Change in policies	15.4	2
Working from home	7.7	1
Furloughed	7.7	1
**If participant reports that their children live with them:**		
Has coronavirus affected your childcare (*n*=18)	No (77.8)	14

**Table 4:** COVID-19 Activity Restrictions (N=33), %(n)

	Never	Rarely	Sometimes	Often
In the past month, how often have you…				
Gone to other social groups like AA/NA	100 (33)	0	0	0
Attended parties	97.0 (32)	3.0 (1)	0	0
Gone to church	90.9 (30)	6.1 (2)	3.0 (1)	0
Visited with friends at their home	78.8 (26)	12.1 (4)	3.0 (1)	6.1 (2)
Visited with a family member at their home	45.5 (15)	21.2 (7)	21.2 (7)	12.1 (4)
Gone to Walmart or somewhere like that	12.1 (4)	36.4 (12)	30.3 (10)	21.2 (7)
Gone to the grocery store	6.1 (2)	24.2 (8)	42.4 (14)	27.3 (9)
Gone outside for a walk or exercise	18.2 (6)	9.1 (3)	15.2 (5)	57.6 (19)
